# Eccentric Resistance Training in Youth: Perspectives for Long-Term Athletic Development

**DOI:** 10.3390/jfmk4040070

**Published:** 2019-11-28

**Authors:** Benjamin Drury, Sébastien Ratel, Cain C.T. Clark, John F.T. Fernandes, Jason Moran, David G Behm

**Affiliations:** 1 Department of Applied Sport Sciences, Hartpury University, Gloucestershire GL19 3BE, England, UK; john.fernandes@hartpury.ac.uk; 2 Laboratoire des Adaptations Métaboliques à l’Exercice en conditions Physiologiques et Pathologiques (AME2P, EA 3533), Université Clermont Auvergne, F-63000 Clermont-Ferrand, France; sebastien.ratel@uca.fr; 3 Faculty of Health and Life Sciences, Coventry University, Coventry CV1 5RW, England, UK; ad0183@coventry.ac.uk; 4 School of Sport, Rehabilitation and Exercise Sciences, University of Essex, Colchester CO4 3WA, UK; jmorana@essex.ac.uk; 5 School of Human Kinetics and Recreation, Memorial University of Newfoundland, St. John’s Newfoundland and Labrador, A1C 5S7C, Canada; dbehm@mun.ca

**Keywords:** eccentric training, youth athletes, paediatric physiology, landing mechanics, flywheel training, eccentric hamstrings

## Abstract

The purpose of this narrative review is to discuss the role of eccentric resistance training in youth and how this training modality can be utilized within long-term physical development. Current literature on responses to eccentric exercise in youth has demonstrated that potential concerns, such as fatigue and muscle damage, compared to adults are not supported. Considering the importance of resistance training for youth athletes and the benefits of eccentric training in enhancing strength, power, speed, and resistance to injury, its inclusion throughout youth may be warranted. In this review we provide a brief overview of the physiological responses to exercise in youth with specific reference to the different responses to eccentric resistance training between children, adolescents, and adults. Thereafter, we discuss the importance of ensuring that force absorption qualities are trained throughout youth and how these may be influenced by growth and maturation. In particular, we propose practical methods on how eccentric resistance training methods can be implemented in youth via the inclusion of efficient landing mechanics, eccentric hamstrings strengthening and flywheel inertia training. This article proposes that the use of eccentric resistance training in youth should be considered a necessity to help develop both physical qualities that underpin sporting performance, as well as reducing injury risk. However, as with any other training modality implemented within youth, careful consideration should be given in accordance with an individual’s maturity status, training history and technical competency as well as being underpinned by current long-term physical development guidelines.

## 1. Introduction

The physical development of youth athletes is an important component in promoting the qualities that underpin athletic performance [1]. A number of previous position statements provide practitioners working with youth athletes, training guidelines that support the long-term athletic development (LTAD) of the individual [2,3,4,5]. The overarching consensus of these statements is largely based upon the principles of the youth physical development (YPD) model, which offers a comprehensive approach to the development of both young males and females via the integration and prioritization of training different physical qualities throughout childhood, and beyond [6]. The rationale of this approach is underpinned by the influence of an individual’s maturity status on physical and performance capacities in youth [7]. Central to the YPD model is the development of muscular strength, which should be targeted throughout youth, for both males and females, due to its underpinning of performance capabilities. Indeed, the importance of muscular strength in supporting young athletes’ performances has been demonstrated on tasks such as change of direction [8,9], vertical jump [9,10,11], leg stiffness [11], sprint ability [9,12] and balance [13]. Accordingly, as muscular strength is an integral component of youth strength and conditioning programs for performance enhancement [2] and also for reducing the risk of sport-related injuries [14], training modalities that can further develop this component are necessary.

Developing muscular strength via the inclusion of resistance training (RT) methods is pivotal [15]. The use of RT for young athletes in developing strength and power qualities that support athletic performance has been strongly advocated [16] and the physiological adaptations underpinning such benefits discussed [17]. Similarly, a large body of evidence exists highlighting the efficacy of using RT methods to enhance the physical capabilities of youth athletes in both male [18] and female [19] populations. For example, recent meta-analyses on the use of RT in youth athletes have provided important information detailing the role of RT variables [20] as well as the different methods that can be used to improve strength, power and speed [21]. Practically, these studies have highlighted the necessity of a variety of training methods required to elicit further performance gains in individuals who are more mature and have a relatively longer training history. Therefore, for practitioners working with youth athletes, the knowledge of RT modalities that could be used for the development of physical qualities and reduce injury risk is beneficial.

Previously, the benefits of utilizing eccentric resistance training (ERT) to enhance performance has been discussed in detail [22]. Furthermore, the physiological responses and chronic adaptations to eccentric training have been outlined [23,24]. However, although the reported benefits of applying ERT methods to improve aspects such as strength, power, speed, change of direction, hypertrophy and injury resistance in adult athletic/trained individuals [25,26,27,28,29,30,31,32,33,34,35,36,37,38,39,40,41,42,43,44,45,46,47,48,49,50,51,52,53,54,55,56,57,58,59], limited information exists pertaining to its practical application within youth athletes. This is somewhat surprising given that specifically targeting the aforementioned physical qualities throughout youth are widely recommended [2]. Since athletic movements in youth will normally include performance of tasks such as changes of direction, deceleration, landing, and hopping, exposure to eccentric muscle actions may already occur within the demands of sport or even playground activities. Reasons for the paucity of research or application within youth are unknown. Though, it may be postulated that factors that are associated with eccentric exercise such as muscle damage [60], muscle strains [61] and the more detailed application of ERT in higher-trained athletes [62] may contribute to this dearth of research concerning applied training modalities. However, it has been recommended that the execution of eccentric training should be emphasized during later LTAD stages in youth [63]. Importantly, practitioners working with youth athletes should understand the acute responses to eccentric exercise as well as the necessity to include ERT modalities throughout maturation stages to enhance physical performance and injury prevention strategies. Therefore, the aim of this article is to provide an overview of the physiological responses to ERT in youth, and to discuss the potential applications of ERT within LTAD. It is hoped that this knowledge can provide practitioners working with youth athletes’ greater awareness of the benefits of including ERT as well as potential training prescription and programming concepts.

### 1.1. Terminology

The terms ‘children’ and ‘pre-pubertal’ refer to girls and boys that are within the pre-peak height velocity maturation stage (pre-PHV), roughly defined as up to the age of 11 for girls and up to the age of 13 for boys. This differs from the terms ‘pubertal’ and ‘adolescent’ which normally include girls aged 12–18 years and boys aged 14–18 years old (circa and post-peak height velocity stages). The use of the word ‘athlete’ will refer to a person who competes in sport.

### 1.2. Literature Search

With no date restrictions, a Boolean logic systematic search from online databases including Google Scholar, PubMed and Web of Sciences was undertaken from which only English language articles were considered. The following terms were searched: ‘eccentric training’, ‘eccentric exercise’, ‘youth athlete’, ‘maturation’, ‘nordic hamstring exercise’, ‘eccentric hamstring strength’, ‘landing kinematics’, ‘augmented eccentric training’, ‘tendon’, ‘fatigue’, ‘resistance training’, ‘flywheel inertia training’, ‘physiological responses’, ‘injury’ ‘muscle damage’. Boolean operators (AND, OR) were used to concentrate the search terms.

## 2. Neuromuscular and Metabolic Responses to Exercise in Youth

Although the benefits of younger individuals utilizing strength and power methods to enhance physical qualities have been widely documented [21], it is also acknowledged that children and adolescents produce lower levels of muscular force than adults, even when normalized to body mass [64,65,66,67,68]. These differences can be attributed to anatomical and physiological changes occurring throughout growth and maturation [69]. Such factors include muscle size, histology, internal and external joint leverage, tendon mechanical properties and the central nervous system [70]. Furthermore, it is acknowledged that children recover quicker from high-intensity exercise than adults and therefore the same training principles may not apply [71]. An understanding and awareness of these different responses to RT in youth compared to adults is important to allow for appropriate prescription as these responses and adaptations underpin the guidelines used to structure resistance training for youth athletes [6].

### 2.1. Fatigue Resistance

From a neuromuscular fatigue perspective, it has been demonstrated that younger individuals are more fatigue resistant than adults [72]. The lesser fatigue in youths compared to adults following exercise has been shown within a range of exercise modalities with pre-pubertal boys displaying a greater maintenance of peak torque than adults following dynamic maximum isokinetic [67,73,74,75,76,77] and isometric [65,78,79] actions of the knee extensors and flexors, maximal isometric exercise of the elbow flexors [68], and isometric exercise of the plantar flexors [64]. The lower reductions in force-generating capacity following resistance exercise-induced fatigue in youth compared to adults have been shown to occur in conjunction with smaller alterations of neuromuscular properties including sarcolemma excitability (i.e., M-wave amplitude), excitation–contraction coupling (i.e., low-to-high frequency tetanic force ratio or low-frequency fatigue), muscle contractility (i.e., high-frequency torque) and muscle oxygenation [63,64,68,71,72,73,78,79,80,81]. Conversely, during submaximal isometric RT, differences in fatigue responses including torque and surface electromyography (EMG) have not been observed between children and men [82,83]. The absence of differences during submaximal actions have been attributed to the likely predominance of slow twitch motor units used during submaximal exercise, which are activated according to the size principle [84]. An explanation for this may be that submaximal intensity fatigue protocols encourage both children and adults to initially recruit a low proportion of their fast twitch motor units, thus removing the inherited age-related differences in fatigue occurring during maximal muscular actions [68]. Lower levels of force decrement observed within a bout of resistance exercise in youths has also been accompanied by faster recovery between bouts. For instance, boys have been shown to require shorter rest intervals to maintain peak torque during multiple sets of isokinetic exercise of the knee extensors when compared to adolescents and adults [67,85,86]. Evidence also points to the faster recovery of agonist muscle activation levels following the cessation of resistance exercise in boys, compared to men, the execution of lower limb isokinetic exercise following both maximal [65,77] and submaximal [83] protocols. Similar findings have also been reported during upper-body resistance training exercise, in which boys and male adolescents achieve significantly greater repetitions than adults during the bench press [87] and chest press [88] exercises when completing the same between-set recovery period.

The lower levels of force produced by children compared to adolescents and adults appears to influence fatigue and recovery responses. Part of these differences have been attributed to the inability of children to voluntarily activate their motor units due to the lower central drive to the motor unit, which affects the resulting force production [70]. Confirmation of reduced voluntary activation (VA) in children compared to adults has been previously demonstrated [79,89,90,91,92] although some studies report no such differences [64,93,94]. Reasons for the discrepancies of these results have been proposed to be due to the sex and the muscle group investigated but also the mechanical conditions of muscle actions, and specifically the length at which the muscle was evaluated [70]. Notwithstanding, the lower VA levels observed in pre-pubertal children have been attributed to the immaturity of the corticospinal pathways in which the development of interneuronal synaptic connections both at the cortex and the spinal cord increases between the ages of 8 and 11 years [95]. However, some children may be unable to perform maximal-force muscle actions due to a lack of experience performing at a high intensity and, hence, discomfort and inexperience can impair their ability to fully activate [73]. It has also been proposed that pre-pubertal children possess a greater proportion of type I fibres than adults [96] and therefore have an inability to produce the high-power outputs that would be seen in more mature individuals who may possess a greater amount of type II muscle fibres. Equally, the higher electromyography threshold reported in both boys [97] and girls [98] compared to their adult counterparts might reflect a delayed and shorter onset of recruitment of type II motor units [99].

The variances in fatigue during exercise may be muscle-dependent as well as being influenced by the muscle-tendon unit (MTU) length. This has been observed during isometric fatiguing actions of the plantar flexor (PF) and knee extensor (KE) muscle groups in which boys fatigued similarly to men with the PF muscles but to a lower extent with the KE muscles than men [80]. With regards to the MTU length, neuromuscular fatigue at optimal and long MTU lengths are lower in children compared to adults, which are mainly accounted for by central fatigue (i.e., a reduction in drive to the muscle), rather than peripheral fatigue (i.e., failure in muscle contractility and excitation–contraction coupling (E–C). Conversely, at short MTU lengths, the differences in neuromuscular fatigue between children and adults are significantly reduced [80]. The lesser performance fatigability and peripheral fatigue at short MTU length could be partly explained by a lower torque level than at optimal length, where greater alterations in peripheral mechanisms (i.e., EC coupling) are observed. The longer exercise duration at short muscle length could also account for the greater central fatigue. These differences point to a specific effect of MTU length in boys and men. Subsequently, it would appear that MTU stiffness plays a role in mediating the force-generating capability during youth [99]. Indeed, it has been suggested that a compliant tendon may act as a mechanical buffer, which could additionally protect the muscles from any extensive damage and subsequent peripheral fatigue [100]. Lastly, the greater decrease in antagonist co-activation of the knee flexors during repeated maximal voluntary isometric contractions in pre-pubertal children may contribute to limit the loss of force of the knee extensors, and therefore to delay fatigue of the agonist muscles [101].

### 2.2. Metabolic Responses

Throughout youth, the metabolic responses to exercise change with growth and maturation [102]. Following resistance and short-term high-intensity exercise, children have been shown to elicit lower post-exercise peak blood lactate concentrations, faster blood lactate clearance rates [67,75,103,104,105,106,107,108,109], better blood acid-base regulation [110], lower phosphocreatine (PCr) depletion, faster PCr resynthesis rates [111,112,113,114] and faster heart rate recovery [67,75,108,115]. Explanations for the differential responses in youth have been attributed to factors such as accelerated blood circulation due to smaller body size [69], lower relative muscle mass [109] and lower reliance on glycolysis [67]. This predisposition of children to be more reliant on aerobic metabolism for energy demands has been proposed to occur due to age-dependent metabolic and hormonal responses to exercise compared to adolescents and adults [116]. Indeed, pre-pubertal children have a faster PCr recovery following exercise than adolescents and adults, suggesting that there is a progressive alteration in muscle oxidative capacity throughout youth [117]. A greater reliance on aerobic metabolism in children is also supported by findings that they possess a higher oxidative enzyme activity [118,119,120,121] and higher mitochondrial volume density than adults [122]. Due to these physiological predispositions it has been suggested that pre-pubertal children have a similar metabolic profile to that of well-trained endurance adult athletes due to their greater reliance on oxidative metabolism [123]. For example, recent work has shown that pre-pubertal children have a similar net contribution of energy derived from aerobic metabolism, a similar rate of fatigue (i.e., power loss) resulting from short-term high-intensity exercise and comparable post-exercise recovery of oxygen uptake to well-trained adult endurance athletes [115].

### 2.3. Exercise-Induced Muscle Damage

The previously discussed physical differences between youth and adults may also account for the magnitude of exercise-induced muscle damage (EIMD) in response to RT. Therefore, as children and adolescents mature, it is important to consider how EIMD affects muscle function [124]. For instance, it appears that there is a progressive withdrawal of physiological protection against high-intensity exercise-induced fatigue during puberty [125]. Subsequently, as the youth athlete matures and their ability to produce force also increases, there may be a concomitant increase in fatigue and muscle damage responses. Potential magnified responses should be particularly reviewed for eccentric exercise due to the high levels of muscle damage that is caused by this type of movement [60,126]. The muscle damage resulting from eccentric exercise is commonly indicated by decreases in muscle function, delayed onset muscle soreness (DOMS), and increased muscle enzymes and proteins, such as creatine kinase (CK) and myoglobin (Mb) in the blood, respectively [127,128]. Moreover, such effects can last for several days’ post-exercise [129]. This is important to consider as EIMD can impair the physiological adaptations to training [130] and can subsequently influence the prescription of training stimuli [131]. The negative consequences EIMD may have on training exposure in youth is important to consider as higher resistance training frequencies are associated with increased performance improvements in youth athletes [132]. In particular, factors such as these should be planned for in youth to ensure that they can be integrated within the required periodization plan to enhance athletic development [133]. Moreover, prescription of ERT within an individuals’ LTAD plan needs to consider improving physical qualities, but also reducing injury risk [134,135,136]. For that reason, the suggested inclusion of eccentric training during LTAD [63] requires careful deliberation.

It has been reported that children experience less EIMD than both adolescents and adults (Table 1). For instance, after eccentric exercise of the elbow flexor muscles, an age-dependent effect has been observed between children, adolescents, and adults in both females [137] and males [138]. Results of these studies reported reductions in maximal concentric strength of the elbow flexors, range of motion (ROM), muscle soreness, plasma CK, and Mb concentrations were reported to be lower in children than both adolescents and adults, and adolescents lower than adults. Moreover, following maximal eccentric voluntary muscle actions of the knee extensors in which relative total work completed was similar, men experienced more severe muscle damage than boys [139]. This was manifested through compromised muscle function (i.e., reduced eccentric, concentric and isometric peak torque) for several days in men whist children demonstrated no such changes. Similarly, greater increases in muscle damage markers (i.e., DOMS, ROM, CK) lasted up to 96 h for men whilst, in boys, these only persisted for up to 48 h. Differences in DOMS has also been reported in the upper-body too. For example, following completion of an augmented eccentric machine chest press exercise adults reported higher levels of soreness than children 48 h’ post-exercise [140]. A similar augmented eccentric load was also used during the leg curl exercise in females in which CK measured one-, two-, three- and four-days post exercise was higher in adult females compared to female children [141]. Separately, EIMD protocols that have included high volume repetitive jumping, which subsequently expose participants to high landing forces and the associated eccentric control of these actions [142], have again demonstrated that children experience lower levels of muscle damage compared to adults [143,144,145]. Moreover, a number of other studies have reported lower symptoms of DOMS between younger individuals compared to adults following exercise modes of RT [146,147], aerobic exercise [148] and downhill running [149].

### 2.4. Repeated Bout Effect

Performing unaccustomed exercise results in a number of muscle damage responses that can be considered symptomatic (e.g., force loss, muscle soreness), systemic (e.g., increased circulating muscle proteins), or histologic (e.g., myofibrillar disruptions) [150]. Skeletal muscle possesses an intrinsic mechanism that reacts to EIMD via providing an adaptive response to subsequent EIMD stimuli (see [150] for more details). This phenomenon is known as the repeated bout effect (RBE) which has primarily been investigated following eccentric muscle actions due to its greater tendency to elicit a muscle damage response [151]. For example, it is well established that the first bout of eccentric-only exercise protects the respective muscle from further muscle damage during subsequent exercise sessions [152]. Indeed, attenuation of muscle damage markers during the second bout of exercise that is completed within several weeks of the initial bout has been evidenced [153]. Mechanisms explaining this improved response may include neural, muscle-tendon complex behaviours, extracellular matrix structural remodelling and a modified inflammatory response [150]. Although the specific mechanisms that are responsible for the RBE are unclear, it would appear that the RBE is multifactorial which is likely to be highly muscle and exercise specific [154]. The beneficial effects conveyed by the RBE is characterized by factors such as faster recovery of muscle strength and ROM, reduced increases in muscle proteins in the blood, and less significant development of swelling and muscle soreness [152,155].

Although muscle damage following eccentric exercise is lower in children and adolescents compared to adults, the RBE does occur in all populations, although to different magnitudes. The lower protective effect observed in the younger groups might be due to the lower levels of muscle damage evident following eccentric exercise. That is, a greater initial degree of muscle damage might induce a greater RBE. The magnitude of the RBE has been shown to correspond with the intensity of the first eccentric exercise bout, such that the higher the intensity, the greater the level of protection provided [156]. For example, after plyometric exercise an RBE was evident for all symptoms examined in men, but only for muscle soreness in boys [145]. Likewise, following plyometric exercise in children, young adults and elderly populations, isometric torque recovery was significantly greater after the second bout of plyometric exercise in all groups, but this improvement was accompanied by a higher level of voluntary activation only in young adult males [144]. Since children are less susceptible to eccentric exercise-induced muscle damage than adolescents and young adults, it may be that the RBE of children is not expressed as strongly. However, following eccentric exercise in both male and female pre-pubescent, pubescent youth, and adults, the magnitude of the RBE was similar [137,138].

The above discussed findings indicate that although children tend to experience less EIMD after the initial bout of eccentric exercise, they may potentially have similar adaptability to eccentric exercise as adults. However, it is necessary to highlight that participants in these prior studies were of untrained status and therefore responses may be different for those youth athletes who are engaged with regular resistance training. For example, it has recently been shown that athletes compared to non-athlete’s experienced different responses in which athletes are able to recover quicker despite displaying a greater force decrement following the eccentric bout of exercise [153]. Furthermore, when comparing between resistance-trained and untrained individuals, resistance-trained individuals seem to show less RBE than untrained individuals [157]. Overall, based on current findings it would appear that children and adolescents may adapt to ERT in a similar way as adults. Subsequently, it has been suggested, that an approach of beginning with low-intensity, prior to gradually increasing the intensity and volume based on the progressive overload principal is suitable for both youth and adults [137]. However, further research investigating the RBE following ERT in well-trained youth athletes is warranted.

### 2.5. Eccentric Resistance Training Safety Considerations for Youth Athletes

Current research demonstrates the maturation- and age-dependent effects on neuromuscular fatigue, recovery, and muscle damage in which there is a progressive transient from childhood through to adulthood. Particularly, concerns regarding the increased risk of muscle damage in children and adolescents compared to adults appears to be unfounded with younger individuals experiencing less severe symptoms following exercise. These differences may be explained due to the lower force production capacity of children and adolescents, leading to reduced fatigue and muscle damage symptoms. Furthermore, this may also be explained by the greater total work performed during ERT protocols in adults compared to adolescents and adolescents compared to children [138]. It should also be recognized that populations used in previous studies have largely been of untrained status and, therefore, responses in trained youth may be different. Despite this, current evidence suggests that the inclusion of ERT methods in youth should not cause concern with regard to increased levels of muscle damage, fatigue or injury risk when compared to other resistance training modalities in youth that are conventionally used. Indeed, it has previously been suggested that concerns for performing eccentric exercise in youth may be due to its potential for high muscle force production and potential for predisposing children to a higher risk of muscle injury [158]. However, although such concerns may be alleviated by the information presented in this section, the use of ERT in youth requires a well-structured approach that considers a range of elements such as the individual’s maturity status, training experience, resistance training background, and movement competency. As such, the next section of this article will present the potential applications for ERT in youth and how these may be implemented within practice.

## 3. Implications for Eccentric Resistance Training in Youth

As denoted in Table 1, youths experience less EIMD than adults following exercise. These differences in muscle damage are observed not only in performance of tasks including jumping, running, and conventional RT but also ERT, too. Therefore, although further research is still warranted, concerns regarding the efficacy of using ERT during childhood and adolescence are not currently supported by current evidence. This is important to acknowledge as it is in the authors’ experience that the use of ERT within youth is limited. However, youth athletes engage with movement skills such as landing, sprinting and change of direction (COD), which are key within the YPD model [6]. Thus, youth athletes are likely already exposed to such stimuli that requires high levels of force absorption. More advanced RT methods, such as plyometrics, which incorporate substantial eccentric muscle actions during landing, have been advocated for children and adolescents [21]. It is evident that current recommendations for youth athletes already include training modalities that emphasize eccentric muscle actions. However, we would suggest that a more holistic approach for the inclusion of ERT could be provided to support current RT guidelines for youth [41]. Additionally, the role of ERT for injury prevention throughout youth may be beneficial as during peak height velocity (PHV), rapid growth, and subsequent temporary disruptions in motor control can increase injury risk [159,160,161,162]. Given current recommendations for integrating ERT for injury preventions purposes in youth athletes [163], the inclusion of eccentric muscle actions into youth training may be particularly important. Therefore, the integration of ERT into current LTAD model(s) could have beneficial implications for those working with youths, particularly regarding performance enhancement and injury prevention. Consequently, the inclusion of ERT for the youth athlete is worth consideration.

### 3.1. Landing Mechanics

Tasks such as hopping and jumping are classified as fundamental movement skills in youth [164] and can be considered as the “building blocks” for further, more complex, movements [165]. Such movements are deemed important for athletic performance [166]. To perform these movements effectively the individual requires eccentric capabilities during the landing phase [142] to absorb kinetic energy and large vertical forces that are experienced to preserve the integrity of the lower limbs anatomical structures [167,168]. This is important as the inability to absorb such forces experienced during landing has been identified as a mechanism for lower-extremity injuries [169,170]. In particular, a focus on developing correct technique and force absorption qualities within youth is necessary as it has been demonstrated that maturity stage can promote inefficient kinematic and kinetic factors that are associated with increased injury risk [171]. Indeed, children as young as 10 years old demonstrate “risky” movement patterns during landing tasks [172,173], which include neuromuscular risk factors, such as low knee flexion angle (“stiff landing”) and increased knee valgus [174,175]. Furthermore, both boys and girls displayed a longitudinal increase in external knee abduction moments throughout puberty [176,177]. Therefore, the early inclusion of developing appropriate landing mechanics as an injury prevention strategy should be implemented early to avoid these negative outcomes in order to reduce injury risk and promote long-term physical activity [178,179].

Differences in landing mechanics in youth are also influenced by sex and maturation with females displaying more aberrant landing kinematics compared to males throughout all stages of maturation [176,178,180,181,182,183,184,185,186,187]. These differences between young females and male can also be explained by anatomical aspects including an increase in both Q-angle and joint laxity as well as a decrease in notch width [169]. Such maturational effects are not present to the same extent in young males as improvements in knee valgus scores have actually been found with advancing age and stage of maturation [188,189]. This is further confounded in that compared to females, male youth appear to improve their lower limb control, sagittal plane motion, and landing forces during landing as they mature [190,191,192]. Differences in neuromuscular performance between sexes during and following puberty may contribute to altered biomechanics and resultant forces on the knee. Excessive knee loads, especially in the frontal plane may explain the increased risk of anterior cruciate ligament (ACL) injury in females following puberty and may help identify the optimal time to implement injury prevention programs [176]. Therefore, it is important to ensure that force attenuation during landing is focused upon throughout youth. Although it has been found that a tendency occurs for more mature male players to reduce their knee valgus scores, a high frequency of circa-PHV and post-PHV players still demonstrated moderate and severe knee valgus scores [188]. Additionally, during circa-PHV in young males, increases in inter-limb knee-valgus asymmetries during landing have been observed as well as an increase in normalized landing forces [188,189], which can lead to temporary decrements in motor control and neuromuscular function due to the rapid growth in limb length [193]. Subsequently, it would appear that throughout maturation, despite there being perhaps certain maturity stages that require a specific focus on certain types of training, the correct execution of landing mechanics and the preparation for these tasks are important and an individualized approach for both females and males is required.

Practitioners implementing exercises to improve landing kinematics should carefully consider the prescription of jump-type activities, particularly the volume and intensity of take-off and landing phases to reduce the risk of injury [194]. The increased risk of injury becomes especially apparent as landing forces of approximately 3.5, 8 and 3 times one’s bodyweight have been reported during a single-leg vertical jump, bilateral vertical jump to 50% maximum jump height and single leg hop to 75% maximal horizontal hop distance in youth [172,189,195], respectively. A high ground-reaction force loading rate indicates that an athlete is subjected to high ground-reaction forces upon the initial landing phase, making it difficult to adequately dissipate the forces reaching the knee joint [196]. Practitioners should, therefore, be mindful that greater ground reaction forces, which are present in children compared to adolescents [197,198], may increase risk of injury during landing [199]. For example, performing more intense landing tasks can increase ground reaction forces that negatively influence the frontal plane projection angle (FPPA) [193,200]. Consequently, it would appear that the ability to absorb ground reaction forces may be challenged due to the greater increases in landing forces that are required with increasing drop height during drop landing and drop jump tasks [201,202,203,204]. It is thus necessary to develop the ability to effectively absorb ground reaction forces upon landing via developing appropriate eccentric strength qualities to help reduce biomechanical risk factors such as increased knee valgus and joint moments. Indeed, strength training has been reported to positively change FPPA during landing [205]. In particular, eccentric muscle strength of the lower limbs has been shown to positively influence landing kinematics [206,207,208,209,210,211,212]. In accordance with these findings it is thus advisable for youth athletes to develop both technical proficiency and eccentric strength qualities to assist development of landing mechanics that may aid in the reduction of injury risk.

**Figure 1:**
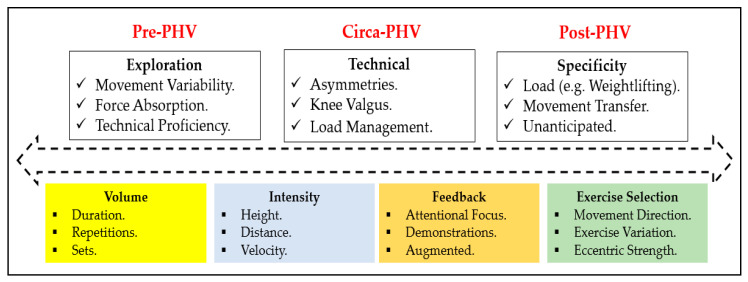
Example conceptual model for the development of landing kinematics throughout youth considering maturity status, injury risk factors and acute training variables. Pre-PHV = pre-peak height velocity. Circa-PHV = circa-peak height velocity. Post-PHV = post-peak height velocity.

As provided in Figure 1, there are a number of approaches that can be proposed to develop force absorption ability throughout youth that should consider aspects of exercise volume, intensity, movement exploration and complexity. For example, the inclusion of activities such as parkour (Exploration) earlier in the child’s development may a provide varied and diversified training programme [213] that provides individuals with the opportunity to sample different movements to manage forces [214,215]. Previously, it has been revealed that parkour participants are more effective at lowering the kinetic landing variables that are associated with a higher injury risk in comparison to recreationally trained individuals [216]. An approach that promotes movement exploration may be helpful in reducing future injury risk due to issues surrounding sport-specialized youth athletes landing biomechanics [217] and the reported anterior knee pain disorders compared to multi-sport athletes [218]. As the individual reaches PHV, further progressions could be included by integrating exercises that have a unilateral landing (Technical) focus in order to aid in the reduction of any asymmetries that may occur during this stage of maturation [188,219,220]. During this stage, increasing the complexity of the jump-landing can be achieved via increasing the jump velocity (intensity) which will subsequently challenge landing kinematics via achieving a greater jump height [221]. The inclusion of weightlifting derivatives (Specificity), which have been advocated previously in youth [222,223], could also be included, more specifically at the post-PHV period. Inclusion of weightlifting derivatives, such as the jump shrug, hang power clean and hang high pull, can be also used to improve load absorption characteristics [224,225]. Such an approach would further challenge movement complexity as well as developing concentric neuromuscular power during the propulsive phase of the movement and eccentric force qualities during the landing phase. Based upon previous recommendations to reduce injury risk in youth [226], we would suggest an approach based on participants’ maturation status, exercise variations (variations), utilisation of verbal feedback (feedback) and, finally, exercise dosage (volume). Although limited information exists pertaining to the use of training volumes for landing mechanics in youth, it has been shown that sessions including up to six sets of six repetitions when completing the drop landing exercise have improved landing kinematics in adults [227]. As a result, it may be sensible to build training volume towards this level throughout the stages of youth. This may be achieved via varied exercise selection [226] with a frequency of two to three times per week which has been suggested for plyometric exercises in this population [228].

### 3.2. Eccentric Hamstrings Strength

The inclusion of exercises to increase eccentric strength of the knee flexor muscles is considered necessary to help reducing the injury risk of the hamstrings muscles [229]. Typically, these injuries have been suggested to occur during sprinting mainly in the late swing phase in which the hamstrings are highly activated at longer lengths and therefore creating high levels of stress on the MTU [230,231,232,233]. Exercises such as the Nordic hamstrings exercise (NHE), used to strengthen the knee flexor muscles eccentrically, is deemed important not only from both an injury prevention perspective but for performance too (e.g., sprinting and change of direction) [50,51,52,234]. Furthermore, the NHE has also been shown to positively influence performance measures such as sprint speed, change of direction, and jumping in both youth males and females [235,236]. Yet, caution to the use of the NHE was previously been suggested on the basis that it was considered too intense for young, inexperienced athletes [237]. Despite this, as presented in Table 1, the use of eccentric muscle actions does not appear to place the youth athlete at greater risk of muscle damage than adults. Appropriate NHE prescription as well as other exercises that develop eccentric hamstrings strength [238,239,240] should be permissible in youth. Indeed, the NHE should form part of a holistic injury prevention program within youth to ensure that the hamstrings receive the specialist consideration that they require throughout youth [241]. In support of this, we have recently found eccentric hamstrings strength improvements in male youth soccer players from the age of 10 years [242].

**Figure 2:**
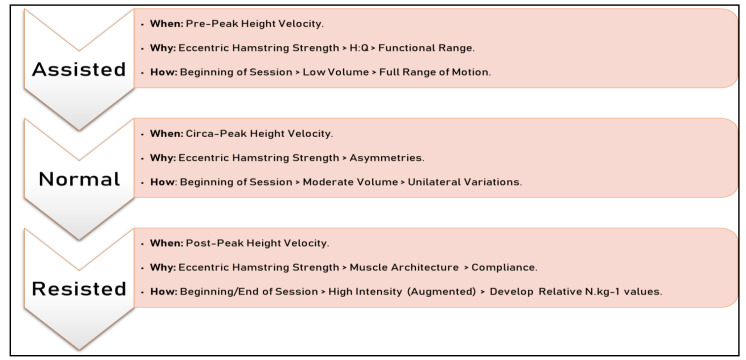
Example progressions for the NHE exercise throughout the different stages of maturation in which the progression of NHE intensity is achieved via assisted and resisted exercises. These progressions are proposed to align with different maturity stages to promote qualities that will aid in the prevention of hamstring injuries.

Inclusion of ERT exercises within an injury prevention program (IPP) is advocated across a number of youth sports [243,244,245,246,247]. Although hamstrings strength has been shown to develop throughout all stages of youth [248,249,250,251,252,253,254,255,256,257], it is important to ensure that it is specifically trained throughout and begun early on in childhood to reduce future risk. This is particularly essential for girls as it has been shown that insufficient hamstrings strength is evident in childhood [190,258]. Moreover, it has been found that girls with reduced hamstrings strength display greater biomechanical ACL injury risk factors during landing actions [259]. Specific development of the medial hamstrings has been instructed in female youth to counteract the external knee valgus moments and knee outward rotation moments [260]. Use of the NHE for females may be particularly helpful considering its reported benefits in reducing ground reaction forces during landing [261] and reducing bilateral hamstrings strength imbalance [262]. Requirements for the development of hamstrings strength also applies to male youth as practitioners have reported that players aged 13–16 years are at the greatest risk of injury and that eccentric hamstrings strength is amongst the most important injury risk factors [263]. This would coincide with PHV in which the greatest rate of growth occurs and has been associated with increased injury risk [160,161,162]. Consequently, it is important to ensure that youth athletes are physically prepared for this accelerated period of growth. This is further evidenced by the reports of decreased hamstrings to quadriceps ratio towards the latter stages of adolescence [248,264] and reduced relative eccentric hamstrings strength scores decreasing at senior level [250]. As provided in Figure 2, eccentric hamstrings strength should be targeted throughout childhood, adolescence and adulthood. Attention needs to focus on areas such as reducing asymmetry throughout the PHV stage and ensuring exercise compliance [265] to help maintain eccentric hamstrings strength levels. Once athletes enter the senior level, training and competition commitments may limit the development of this eccentric hamstrings strength.

Concerns regarding the inclusion of ERT at earlier stages in youth should have been alleviated by the information provided in Section 1 of this article. This is also supported by evidence that increases in hamstrings strength have been shown in basketball players aged 10–12 years following a five-week NHE programme [266]. However, as per previous recommendations, we would also suggest that the NHE should form part of a holistic hamstrings programme [267]. Therefore, although we feel that the inclusion of the NHE in youth should be viewed as a fundamental exercise used to increase eccentric hamstrings strength, other eccentric hamstrings exercises that target both distal and proximal hamstrings regions should be integrated [239] as well as inclusions of concentric [268], isometric [269] and sprinting [270] exercises. These should be completed with correct technique, appropriate progressions and in accordance with current RT guidelines for youth. Additionally, an emphasis during youth should be placed upon developing eccentric hamstrings strength across its full ROM due to the proposition that a shift in the optimum angle of peak torque provided by performing eccentric hamstrings exercises at long muscle lengths reduces hamstrings injury risk [58,271]. Moreover, both male and female senior players possess higher angle specific torque and functional range eccentric knee flexor values compared to their respective youth counterparts [272,273]. Ensuring that exercises target the full range of movement may also aid in the development of the hamstrings muscle architectural properties. This may be because shorter biceps femoris fascicle lengths can increase hamstrings injuries [274]. Considering the positive effects of eccentric exercise on properties such as fascicle length [54,271,274,275,276,277,278,279,280,281,282], as well as the relatively quick reversal of hamstrings muscle fascicle length and strength adaptations [278,279,280], perhaps the regular and structured inclusion of eccentric hamstrings exercises throughout youth could be viewed as a preparatory approach to developing architectural properties of the hamstrings and a foundation of eccentric hamstrings strength that can be maintained and progressed at senior levels. This is because it has been proposed that increases in fascicle length typically occur during the pre-pubescent period, whereas substantial increases in muscle cross-sectional area (CSA) typically occur during the pubescent period [283,284]. As a result, it may be advisable to develop structural properties and strength qualities earlier on in youth via the progression of training volume prior to increasing the intensity of the exercise post-PHV, once sufficient absolute and relative eccentric hamstrings strength has been achieved [285]. Indeed, it has been reported that additional loads are required during exercises such as the NHE in order to promote further eccentric hamstrings strength increases and fascicle length [286]. Subsequently, increases in muscle architecture properties at post-PHV status may also be targeted via performing the NHE either pre or post training to elicit specific morphological adaptations [287].

### 3.3. Flywheel Inertial Training

Typically, during RT, overload provided during the exercise remains constant for both eccentric and concentric portions of the exercise consequently leading to a lower relative load being lifted during the eccentric phase [288]. This is because greater forces are sustained during eccentric muscle actions compared to that of isometric and concentric actions [289,290]. For example, accentuated eccentric loading (AEL), which includes a load during the eccentric phase that is in excess of the concentric load [22] can be incorporated. AEL is considered an advanced training tactic [291]. Accordingly, we would suggest AEL to be included towards the latter stages of adolescence once an appropriate foundation of eccentric strength and resistance training skill competencies has been developed [63]. An alternative method to create eccentric overload in youth that may be appropriate is that of flywheel inertial training (FIT). FIT offers additional resistance throughout the entire ROM via the use of the inertia of a rotating flywheel to provide a greater overall load during coupled concentric and eccentric muscle actions [292]. The use of FIT in adult populations has been reported to provide many benefits including improvements in physiological, physical and performance factors, such as running economy [293], body composition [294], muscle activation [295,296], acute power enhancement [297,298,299,300], muscle architecture [35,301,302,303,304], change of direction [35,48,305] as well as force- and power-related qualities [25,305,306,307,308].

To date, some evidence does exist relating to the benefits of FIT in youth athletes. For example, in youth team sport athletes FIT has shown to improve performance in tasks such as jumping, sprinting, as well as the ability to increase breaking forces during change of direction [47,309,310]. Specifically relating to change of direction (COD), knee extensor eccentric strength has been associated with improved deceleration in youth (16.8 years) male soccer players [311]. Such findings could be expected considering that faster COD performance has been associated with higher levels of braking force application [312,313]. Since during adolescence greater increases in speed [314,315,316] and strength and power qualities [317,318,319,320,321] are observed it is important that the propulsive forces developed to achieve these are also balanced with the appropriate eccentric strength characteristics too. Without such a focus this may place the youth athlete at greater risk of injury as they may not be able to effectively decelerate from the increased running speeds they can achieve. Indeed, it has been shown that performing cutting movements at greater speeds negatively affects lower extremity biomechanics that are associated with ACL injury risk [222,322]. These factors warrant due attention as increases in body mass also accompany changes in maturity status [323]. These combined increases will likely result in greater amounts of momentum being produced. For example, in youth rugby union players it has been shown that maturity status is a significant predictor of momentum [324]. Accordingly, it is important that the youth athlete possesses the sufficient eccentric force qualities to tolerate the force demands from such tasks as COD and deceleration that they will be exposed to not only support performance but to aid in reducing injury risk.

**Figure 3:**
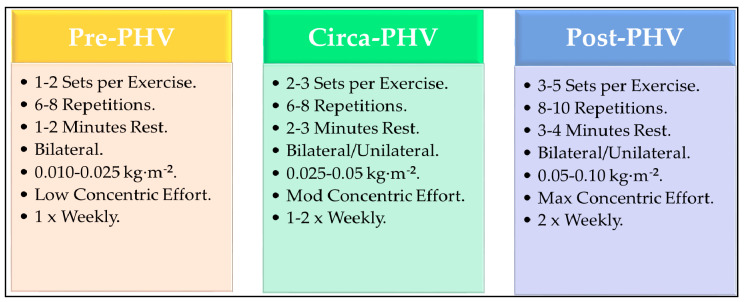
Proposed overview of incorporating FIT methods throughout childhood and adolescence. Please note, different exercises may require different flywheel inertia intensities. Pre-PHV = pre-peak height velocity. Circa-PHV = circa-peak height velocity. Post-PHV = post-peak height velocity. Mod = Moderate. Max = Maximal.

Considering that physical qualities, such as COD, speed and strength, are recommended to be developed throughout all stages of youth [6] as well as the aforementioned benefits of FIT, its implementation within youth may be beneficial. This is appealing since the magnitude of eccentric forces encountered by a flywheel device during an exercise is proportionate to the preceding concentric forces [325]. Such an approach may be appropriate in youth as their lower concentric force capability would result in eccentric forces that would be proportionately lower [326]. In addition, it has been demonstrated that the use of higher flywheel inertias can concomitantly provide eccentric overload via increases of kinetic variables such as negative impulse during the descent phase of a movement [292,327,328]. Therefore, the introduction of low intensity flywheel inertia wheels may represent an appropriate starting point for training progressions along with lower training volumes prior to gradually increasing the load, volume and frequency to that which has been currently reported for FIT in adult populations [25,35,292,302,305] and which reflects current youth resistance training guidelines [17]. Such an approach is provided in Figure 3. Furthermore, since muscle damage following FIT have been shown to reflect those routinely reported following eccentric exercise [329], it important that as the individual reaches post-PHV the inclusion of eccentric overload training within the micro-cycle is further carefully deliberated. The potential greater increases in concentric force production during FIT that would be expected as a product of growth, maturation and training exposure will subsequently result in higher eccentric overload too, which may subsequently heighten fatigue and muscle damage responses that are observed in adults.

## 4. Other Programming Considerations

An important consideration for injury prevention throughout youth is the occurrence of conditions such as tendinopathies and how ERT may be able to aid with this. Prevention of these injuries should be targeted in children and adolescents as it is now understood that these may occur earlier than originally thought [330]. An early approach is necessary as knee-related pain injuries such as these can have a negative impact on athletes’ future performance and career [331]. The risk for tendinopathy injuries throughout youth would appear to be contributed to by factors such as overuse due to the loading of tendons during sports or vigorous activity [332,333]. This is particularly prudent for the youth athlete as it has been shown that sport specific tendon adaptations such as greater tendon thickness occur in adolescent athletes compared to non-athletes [334,335]. Moreover, a higher prevalence of structural intratendinous changes have been observed in adolescent athletes with patellar tendinopathy symptoms than those without [336]. In addition, intratendinous alterations that were associated with tendinopathies have been reported in adolescent youth athletes compared to recreationally active controls [337]. However, such issues may not only be impacted by training activity but also impacted by growth and maturation processes due to increases in aspects such as moment arm lengths and muscle activation, which leads to a disproportionate increase of muscle strength [338]. Indeed, it has been observed that an imbalance exists between the development of the muscle and tendon in which the greater adaptations and development of the mechanical properties of the tendon occur towards the end stages of adolescence [339,340,341]. Furthermore, it has been demonstrated that adaptations to training during the earlier stages of youth result in increases in strength but not tendon stiffness [342]. Considering that adaptations of tendon properties to resistance exercise are slower than those of muscle strength [343,344] this may cause an imbalanced adaptation of the muscle and tendon and risk overload and tendon-related injuries [345]. Support for this concept within youth has recently been shown as high levels of tendon strain in adolescent basketball athletes were associated with micro-morphological deterioration of the collagenous network in the proximal patellar tendon, a frequent site affected by tendinopathy [346]. Furthermore, adolescent athletes have been found to reach greater strain magnitudes compared to non-athlete controls indicating an increased mechanical demand for the patellar tendon [347]. Therefore, in light of the aforementioned, specific training that increases tendon stiffness and facilitates a balanced adaptation between muscle and tendon might be important [345,346,347].

In youth it has been proposed that a combination of growth and loading could act as a ‘‘dual’’ stimulus for tendon growth and improvement of its material properties, increasing the tendon’s stiffness throughout maturation [348]. Furthermore, it has been demonstrated that tendon growth from RT, even in pre-pubertal children can occur [349]. It is plausible that training modalities, such as ERT, may be efficacious in not only enhancing force absorption qualities in youth but also reducing tendinopathies by reducing tendon strain via development of the mechanical properties of the tendon. For example, eccentric muscle actions have been shown to positively influence the mechanical properties of the tendon including CSA and stiffness in adult males [350,351,352]. Indeed, the efficacy of using eccentric training in treating tendinopathies has been provided previously in adults [353,354]. The use of ERT may provide a favourable modality as morphological adaptations and mechanical properties of the tendon respond more positively to high action intensities (≥85% isometric muscle voluntary action) and long action durations (≥3 s;) [355,356,357]. Indeed, the use of ERT modalities such as FIT has been shown to positively influence the mechanical properties of both the Achilles and patellar tendons enabling them to be more resistant to deformation post-exercise in adult males [358,359]. Also, six weeks of FIT leg press exercise in adult males suffering with chronic patellar tendinopathy improved tendon pain symptoms as well as strength and neuromuscular activation [360]. Interestingly, the inclusion of FIT in adults who are at risk of patellar tendinopathy have shown it to be appropriate during the in-season of basketball and volleyball players in which no complaints regarding patellar tendon pain were provided as well as displaying improvements in lower limb muscle power [361]. Therefore, training modalities that can provide an increase in tendon stiffness that as well as the force generating capacity of the neuromuscular system may be efficacious to help protect against increased strain during maximum muscle actions [345]. As a result, a potential approach in reducing tendinopathy issues throughout youth, particularly patellar tendinopathy, may benefit from a combination of eccentric strength and force absorption approaches that involve (1) development of effective landing mechanics qualities that help reduce joint moments and ground reaction forces (Figure 1); (2) use of ERT methods such as the FIT to aid in the potential development of the MTU (Figure 3); and (3) increasing eccentric muscular strength to further support landing kinematics. However, further research is required to investigate these areas to support this in youth.

## 5. Conclusions

The inclusion of ERT throughout youth can be incorporated within a well-designed LTAD program that follows current proposed guidelines [6]. However, it should be acknowledged that current research within ERT for youth athletes is its infancy and areas such as training intensities, training volumes, recovery periods and its effects on performance tasks and injury prevention require further investigation. Implementing ERT should be considered as part of a holistic athletic development training programme within youth that should begin during the pre-pubescent stage and progressed throughout all stages of maturation taking into account the individuals technical proficiency, training history, maturity status, and current physical qualities. Initial approaches to the inclusion of ERT may begin within an integrated injury prevention warm up similar to those injury prevention program commonly used for youth but to also ensure a balanced emphasis quality such as landing kinematics, eccentric hamstrings strength, deceleration and COD ability, neuromuscular strength and tendon mechanical properties. Thereafter, specific consideration is required during PHV when injury risk may increase due to maturation-related processes in both male and female adolescents. Once the athlete reaches post-PHV status, further specificity can be provided to elicit greater adaptations due to the athletes’ training history and increased benefits observed from RT as this stage [18,19,69]. However, considering that during youth there is a progressive increase of exercise-induced fatigue [125], the inclusion of ERT at this stage may also accompany increased levels of muscle damage symptoms and therefore this should be planned and educated to the youth athlete. Furthermore, we would encourage that recent articles published on the implementation of eccentric training [62,362] act as a point of reference for youth athletes reaching senior levels of performance that is underpinned by a sufficient training history of ERT throughout youth as presented in this article.

Although there may be concerns with regard to the introduction of ERT in youth athletes compared to adults due to factors such as increased risk of fatigue, muscle damage or injury, current evidence would not support these assumptions. Therefore, concomitant to the physiological demands that youth individuals will be exposed to within their sport, it is important that eccentric force qualities are developed throughout youth and not seen as an advanced RT modality. Indeed, in youth, the use of ERT can be viewed as developing aspects such as force absorption/attenuation during tasks such as landing/COD, reducing strength ratios to reduce injury risk as well as potentially acting as a structural mechanism to develop tendon mechanical properties. Importantly, the inclusion of ERT in youth should be seen as part of a holistic LTAD programme that support the development of physical qualities encouraged within LTAD models such as strength, speed, agility, and other factors. Furthermore, throughout childhood and adolescence the use of ERT should be considered a preparatory approach to sufficiently prepare athletes for the demands of elite performance levels and the more intense and specific eccentric training methods that may be trained throughout this period. To improve the current literature, future work should identify the effects of youth eccentric training with regards to training intensity, volumes, recovery periods and its effect on injury prevention in youth. Until such work has been produced, ERT in youth should implemented on an individual approach with low dosages and small progressions in volume and intensity. However, it would appear that the inclusion of ERT in youth may confer numerous benefits and so practitioners working with this population should contemplate its inclusion within LTAD.

## Figures and Tables

**Table 1:** Studies assessing fatigue and exercise induced muscle damage following resistance training between children, adolescents and adults.

Study	Age (years)	Sex	Exercise Protocol	Selected Measurements	Outcome
[137] Lin et al. (2018)	C: 9–10 (*n* 13) Ad: 14–15 (*n* 13) A: 20–24 (*n* 13)	F	5 × 6 reps of eccentric elbow flexors. Dumbbell weight set at 60% iMVC. 2 min rest between sets.	MVC, DOMS, CK, Mb, ROM, MPS.	A > Ad > C
[139] Deli et al. (2017)	C: 11.0 ± 0.2 (*n* 11) A: 35.3 ± 2.2 (*n* 15)	M	5 × 15 reps of eccentric knee extensors. Dynamometer Set at 60° s^−1^. 2 min rest between sets.	MVC, DOMS, CK and ROM.	A > C
[143] Lazaridis et al. (2018)	C: 10 ± 0.7 (*n* 13) A: 25.3 ± 3.3 (*n* 13)	M	10 × 10 reps of CMJ. 30 s rest between sets.	iMVC, DJ, EMG, K_stiffness_ and RPE.	A > C
[140] dos Santos et al. (2016)	C: 11.3 ± 0.82 (*n* 10) A: 24.5 ± 5.58 (*n* 10)	M + F	5 × 15 reps of eccentric machine chest press. Load set at 110% of 10RM concentric chest press. 3 min rest between sets.	DOMS.	NSD
[138] Chen et al. (2014)	C: 9.4 ± 0.5 (*n* 13) Ad: 14.3 ± 0.4 (*n* 13) A: 22.6 ± 2.0 (*n* 13)	M	5 × 6 reps of eccentric elbow flexors. Dynamometer set at 90^o^ s−^1^. 2 min rest between sets.	MVC, DOMS, CK, Mb, ROM and MPS.	A > Ad > C
[144] Gorianovas et al. (2013)	C: 11.8 ± 0.9 (*n* 11) A: 20.8 ± 1.9 (*n* 11) E: 63.2 ± 3.6 (*n* 11)	M	100 drop jumps. DJ box height set at 0.5 m. 30 s rest between reps.	iMVC, LFF, VA, DJ Height, DOMS, CK.	A > E > C
[146] Pullinen et al. (2011)	A: 31 ± 7 (*n* 8) Ad: 14 ± 0 (*n* 8)	M	3 × sets until exhaustion of concentric knee extensors. Load set at 40% of 1RM bilateral knee extension. 4 min rest between sets.	iMVC, CK, EMG, HR.	A > Ad
[145] Marginson et al. (2005)	C: 9.9 ± 0.3 (*n* 10) A: 22.2 ± 2.7 (*n* 10)	M	8 × 10 reps of CMJ. 1 min rest between sets.	iMVC, DOMS, CMJ, SJ.	A > C
[141] Arnett et al. (2000)	C: 10.5 ± 1.1 (*n* 15) A: 23.4 ± 6.9 (*n* 15) E: 59.4 ± 10.9 (*n* 10)	F	6 × 10 Reps of eccentric leg curl exercise Load Set at 110% 1RM concentric leg curl. 1 min rest between sets.	CK.	A > E > C
[148] Duarte et al. (1999)	Ad: 13.0 ± 0.5 (*n* 10) Ad: 13.2 ± 0.7 (*n* 10)	M	Box step up and down until exhaustion. Tempo Set at 1:1 (1 s up – 1 s down) vs 1:2.	iMVC, DOMS and CK.	1:2 > 1:1
[147] Soares et al. (1996)	C: 12.1 ± 0.2 (*n* 10) A: 28.3 ± 3.5 (*n* 10)	M	5 Sets × 80% 1RM concentric bench press until exhaustion. 90 s rest between sets.	iMVC, DOMS and CK.	A > C
[149] Webber et al. (1988)	C: 10.4 ± 0.3 (*n* 16) A: 27.1 ± 0.87 (*n* 15)	M + F	30 min downhill running @ 10% gradient.	DOMS and CK.	NSD

**C:** Children; **Ad:** Adolescents; **A:** Adults; **E:** Elderly; **M:** Male; **F:** Female; **Reps:** Repetitions. **MVC:** Maximal voluntary contraction; **iMVC:** Isometric maximal voluntary contraction; **CMJ:** Countermovement jump; **DJ:** Drop Jump; **DOMS:** Delayed onset muscle soreness; **EMG:** Electromyography activity; **CK:** Creatine kinase; **Mb:** Myoglobin; **RM:** repetition maximum strength; **LFF:** Low frequency fatigue; **HR:** Heart rate; **ROM:** Range of motion; **VA:** Voluntary; **NSD:** No significant difference.
